# Target tumor microenvironment by innate T cells

**DOI:** 10.3389/fimmu.2022.999549

**Published:** 2022-10-06

**Authors:** Yan-Ruide Li, Matthew Wilson, Lili Yang

**Affiliations:** ^1^ Department of Microbiology, Immunology & Molecular Genetics, University of California Los Angeles, Los Angeles, CA, United States; ^2^ Molecular Biology Institute, University of California Los Angeles, Los Angeles, CA, United States; ^3^ Eli and Edythe Broad Center of Regenerative Medicine and Stem Cell Research, University of California Los Angeles, Los Angeles, CA, United States; ^4^ Jonsson Comprehensive Cancer Center, David Geffen School of Medicine, University of California Los Angeles, Los Angeles, CA, United States

**Keywords:** tumor microenvironment (TME), tumor-associated macrophage (TAM), myeloid-derived suppressor cell (MDSC), innate T cell, invariant natural killer T (iNKT) cell, mucosal-associated invariant T (MAIT) cell, gamma delta T (γδT) cell, cell-based immunotherapy

## Abstract

The immunosuppressive tumor microenvironment (TME) remains one of the most prevailing barriers obstructing the implementation of effective immunotherapy against solid-state cancers. Eminently composed of immunosuppressive tumor associated macrophages (TAMs) and myeloid-derived suppressor cells (MDSCs) among others, the TME attenuates the effects of immune checkpoint blockade and adoptive cell therapies, mandating a novel therapy capable of TME remediation. In this review we explore the potential of three innate-like T cell subsets, invariant natural killer T (iNKT), mucosal-associated invariant T (MAIT) cells, and gamma delta T (γδT) cells, that display an intrinsic anti-TAM/MDSC capacity. Exhibiting both innate and adaptive properties, innate-like T cell types express a subset-specific TCR with distinct recombination, morphology, and target cell recognition, further supplemented by a variety of NK activating receptors. Both NK activating receptor and TCR activation result in effector cell cytotoxicity against targeted immunosuppressive cells for TME remediation. In addition, innate-like T cells showcase moderate levels of tumor cell killing, providing dual antitumor and anti-TAM/MDSC function. This latent antitumor capacity can be further bolstered by chimeric antigen receptor (CAR) engineering for recognition of tumor specific antigens to enhance antitumor targeting. In contrast with established CAR-T cell therapies, adoption of these innate-like cell types provides an enhanced safety profile without the risk of graft versus host disease (GvHD), due to their non-recognition of mismatched major histocompatibility complex (MHC) molecules, for use as widely accessible, allogeneic “off-the-shelf” cancer immunotherapy.

## Introduction

**Figure 1:**
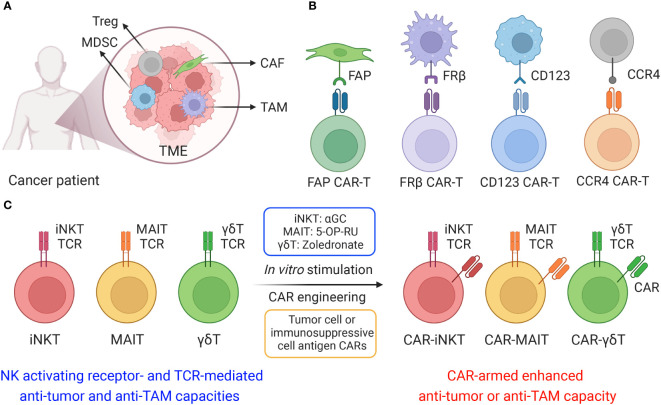
Cell-based therapy to target immunosuppressive cells in solid tumor microenvironment (TME). **(A)** The TME is composed of a heterogeneous milieu of tumor and immune cells. Various immunosuppressive cells such as tumor-associated macrophages (TAMs), myeloid-derived suppressor cells (MDSCs), cancer-associated fibroblasts (CAFs) and T regulatory cells, suppress or reprogram the antitumor immune response. **(B)** Different chimeric antigen receptors (CARs) were utilized to generate CAR-T cells, including fibroblast activation protein (FAP) CAR, Folate receptor β (FRβ) CAR, CD123 CAR and C-C motif chemokine receptor 4 (CCR4) CAR. **(C)** Innate T cells, including invariant natural killer T (iNKT) cells, mucosal-associated invariant T (MAIT) cells, and gamma delta T (γδT) cells, efficiently target immunosuppressive cells, and these innate T cells could be further engineered with CARs and achieve both tumor cell and immunosuppressive cell elimination.

For solid state cancers in particular, the development of a localized tumor microenvironment (TME) has been associated with disease progression, facilitating resistance against targeted immunotherapies. A wide array of cells including tumor-associated macrophages (TAMs), myeloid-derived suppressor cells (MDSCs), cancer-associated fibroblasts (CAFs) and T regulatory cells aggregate within the TME and dampen effector cell response (**Figure 1A**) (1, 2). These immunosuppressive cells have been observed to attenuate immune cell antitumor immunity and promote tumor growth and metastasis (3). Inhibition of T cell-mediated antitumor capacity develops through upregulation of immune checkpoint ligands, such as programmed death-ligand 1 and 2 (PD-L1 and PD-L2), and secretion of immunosuppressive factors, such as transforming growth factor-β (TGF-β), tumor necrosis factor-α (TNF-α), IL-10 and CCL-22 (4). Furthermore, production of pro-angiogenic cytokines and growth factors, including ornithine, TGF-β, vascular endothelial growth factor (VEGF), basic fibroblast growth factor (bFGF) and colony stimulating factor 1 (CSF1), provides nutrient factors that promote tumor angiogenesis and vessel co-option wherein tumor cells hijack the existing patient vasculature, therefore enhancing tumor progression (4, 5).

In order to restore TME-dampened antitumor capacity, therapeutics targeting associated immunosuppressive cells, especially TAMs and MDSCs, to modulate the solid TME have gained traction as an attractive avenue for cancer immunotherapy. One prominent method employs CCR2 antagonism to block recruitment and infiltration of immunosuppressive monocytes/macrophages to the tumor site (6–8). More direct approaches utilize a variety of TAM depleting agents, including clodronate-liposome, melittin-based pro-apoptotic peptide, and mannose-conjugated nanoparticles, to reduce their impact within the TME (9–11). Other approaches harness antineoplastic agents such as natural compound baicalin, paclitaxel, cyclophosphamide, gemcitabine, targeted nanocarrier delivering M1-polarizing transcription factor mRNAs, and CD163-targeted corosolic acid-containing liposomes to reprogram TAMs and restore their proinflammatory phenotype (12–17). Despite the preliminary successes of these immunotherapeutic agents, their transient retention period within the solid TME greatly restricts their interventive potential. These limitations call for a novel therapeutic that persists within the TME for sustained suppression or remediation of immunosuppressive cellular activity.

Recently studies have investigated the potential for cell-based immunotherapy, especially chimeric antigen receptor (CAR)-engineered T (CAR-T) cell therapy, to target the TME. Various CARs, such as folate receptor β (FRβ)-, fibroblast activation protein (FAP)-, CD123-, CCR4- and VEGFR-2-targeting CARs, have been applied to eliminate immunosuppressive cells in the TME with promising effect (**Figure 1B**) (18, 19). FRβ-targeting CAR-T cells have shown the capacity to deplete FRβ^+^ TAMs, delaying tumor progression and prolonging survival in mice (20); FAP is a membrane protease highly expressed on CAFs, FAP-targeting CAR-T cells have been developed to target CAFs in multiple solid tumors, such as mesothelioma, lung and pancreatic cancers (21, 22); The upregulation of CD123 on myelodysplastic syndrome (MDS) clones as well as MDSCs provides a compelling rationale for targeting CD123 antigen by monoclonal antibodies and CD123-targeting CAR-T cells in the immunosuppressive TME of MDS patients (23, 24); CCR4 is highly expressed in T cell malignancies as well as in CD4^+^CD25^+^Foxp3^+^ T regulatory cells, CCR4-targeting CAR-T cells displayed powerful cytotoxicity against a wide spectrum of aberrant T cells, including adult T cell leukemia/lymphoma (ATL), cutaneous T cell lymphoma (CTCL), and anaplastic large cell lymphoma (ALCL) (25, 26).

Innate T cells, including invariant natural killer T (iNKT) cells, mucosal-associated invariant T (MAIT) cells, and gamma delta T (γδT) cells, exhibit intrinsic anti-TAM capacity, and are potent models for CAR-engineering to achieve dual elimination of tumor cells and TAMs (**Figure 1C** and **Table 1**) (27, 28). In this review, we summarized the potential of innate T cell-based therapy for targeting the TME, introducing the immunosuppressive cell-targeting capacity of iNKT, MAIT and γδT cells and reviewing their genetic engineering, preclinical application and translational potential in cancer immunotherapy.

**Table 1:** TCR Comparison Between Different T Cell Types.

T cell type	TCR repertoire	Restriction reactivity	Recognized antigen	Flow cytometry staining antibody (clone) or tetramer
Conventional αβ T	Highly diverse αβ TCRs	MHC-I (for CD8^+^ T cells) and MHC-II (for CD4^+^ T cells)	Peptide antigens	Anti-human TCR α/β (IP26); anti-mouse TCR β chain (H57-597)
Invariant natural killer T (iNKT)	Invariant TCR α chain (Vα14-Jα18 in mice or Vα24-Jα18 in humans) and restricted diverse TCR β chain (mainly Vβ11)	CD1d	Glycolipid antigens (e.g., αGC)	anti-human TCR Vα24-Jα18 (6B11); anti-human TCR Vβ11 (C21); human CD1d/α-GalCer tetramer; mouse CD1d/PBS-57 tetramer
Mucosal associated invariant T (MAIT)	Semi-invariant TCR α chain (Vα19-Jα33 in mice or Vα7.2-Jα33 in humans) and restricted diverse TCR β chain	MR1	Metabolic intermediates derived from the riboflavinbiosynthetic pathway (e.g., 5-OP-RU)	Anti-human TCR Vα7.2 (3C10); human and mouse MR1/5-OP-RU tetramer
Gamma delta (γδ) T	Restricted diverse γδ TCRs	Butyrophilin 3A1, CD1d	MHC-I-related proteins T10 and T22 (mice); Phosphorylated metabolites such as microbial HMB-PP or eukaryotic isoprenoid precursor IPP, lipid antigens (humans)	Anti-human TCR γ/δ (B1); anti-human TCR Vδ2 (B6); Anti-mouse TCR γ/δ (GL3, QA20A16)

## Targeting the tumor microenvironment using iNKT cells

Invariant natural killer T (iNKT) cells are an uncommon subset of αβT cells that exhibit features of both innate and adaptive immune responses (29). iNKT cells present with a specific TCR complex that differs from those of conventional αβT cells; mouse iNKT TCR expresses the Vα14-Jα18 chain paired with a limited number of Vβ chains, typically Vβ2, Vβ7 or Vβ8, whereas human iNKT TCR expresses the Vα24-Jα18 chain with limited Vβ chains, predominantly Vβ11 (30–34). This semi-invariant TCR specifically recognizes lipid and glycolipid antigens presented by the class I MHC-like glycoprotein CD1d (29, 35, 36). Specialized development of iNKT cells in the thymus initially follows that of classical αβ T cells but then diverges during the CD4^+^CD8^+^ double positive (DP) stage (37). Positive selection of the iNKT TCR from antigen-loaded CD1d presentation by cortical thymic epithelial cells (TECs) induces expression of innate NK markers (e.g., CD161) and transcription factor PLZF to produce the mature iNKT cytokine profile and phenotype (38). In addition, iNKT cells can also be activated in a TCR-independent manner in response to antigen presenting cell (APC)-derived IL-12 and IL-18 (39, 40). Upon stimulation, iNKT cells acquire cytotoxicity and secrete large quantities of effector cytokines that stimulate downstream activation of other immune effector cells including NK cells, dendritic cells (DCs), and CD4 helper and CD8 cytotoxic T cells (29, 41). Since their powerful antitumor activity remains independent of antigen priming and MHC restrictions, iNKT cells have become a major focus in the development of novel cell-based immunotherapies. Additionally, implementation of iNKT cells as a strategy for cell-based immunotherapy offers several other advantages, such as eliminating the risk of graft versus host disease (GvHD) from lack of MHC engagement as well as ancillary remediation of the TME through cytotoxic killing of CD1d-expressing TAMs and MDSCs (29, 41–43).

**Figure 2:**
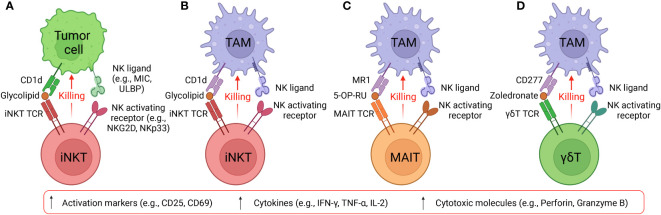
Targeting tumor and immunosuppressive cells by innate T cells. **(A)** Diagram showing iNKT cells target CD1d^+^ tumor cells using NK/TCR double mechanisms. MIC, MHC class I chain-related protein. ULBP, UL16 binding protein. **(B)** Diagram showing iNKT cells target CD1d^+^ TAMs using NK/TCR double mechanisms. **(C)** Diagram showing MAIT cells target CD1d^+^ TAMs using NK/TCR double mechanisms. MR1, major histocompatibility complex, class I-related protein. 5-OP-RU, 5-(2-oxopropylideneamino)-6-D-ribitylaminouracil. 5-OP-RU is a microbial riboflavin-derived antigen and can specifically activate MAIT cells. **(D)** Diagram showing γδT cells target CD1d^+^ TAMs using NK/TCR double mechanisms. Zoledronate is a nitrogen-containing bisphosphonate and can activate γδT cells.

Among macrophages, selective expression of CD1d on TAMs renders iNKT therapy an ideal method for precise disruption of TAM immunosuppression while preserving the pro-inflammatory function of classically activated macrophages (44, 45). CD1d cross-presentation of tumor-derived glycolipids from the surrounding environment enables iNKT cells to eliminate TAMs and dampen their effects (45). Furthermore, iNKT expression of NK activating receptors (e.g., NKG2D, NKp33, NKp40 and DNAM-1) provide a secondary method for these cells to recognize TAMs independently of CD1d presentation (27, 46–48). Recognition of NK receptor ligands on TAMs activates Perforin/Granzyme-mediated lysis and IFN-γ secretion, suppressing the pro-tumoral environment generated by TAMs (46, 49). Previous studies have shown that human peripheral blood mononuclear cell (PBMC)-derived iNKT cells could effectively eliminate M2-polarized macrophages when stimulated with alpha galactosylceramide (α-GalCer or αGC), a synthetic iNKT lipid antigen; iNKT engagement against macrophages was validated through addition of anti-CD1d antibody to block the CD1d/iNKT TCR pathway, which produced diminished killing of CD1d+ M2-polarized macrophages (27, 41). Interestingly, in the absence of αGC, iNKT cells could also kill M2 macrophages albeit at a reduced efficacy (27, 41). This intrinsic killing despite the absence of TCR engagement emerges due to high expression levels of NK activating receptors in iNKT cells for potent NK-mediated cytotoxicity (**Figures 2A, B**) (50, 51). The capacity of iNKT cells to target TAMs through two distinct pathways provides an attractive method to reengineer the tumor microenvironment and stimulate endogenous CD8 cytotoxic T cells and NK cells (52). Another study reported that neuroblastoma TAMs were capable of cross-presenting neuroblastoma-derived endogenous glycosphingolipids from the TME, which could specifically activate iNKT cells and induce iNKT cell-mediated TAM killing (45). The interaction of iNKT cells and CD1d^+^ TAMs within the TME may explain the association between iNKT infiltration with favorable outcome in neuroblastoma and other solid tumors (45). In cases of murine prostate cancer, mouse iNKT cells directly targeted M2-like macrophages through CD1d recognition and engagement of Fas-FasL mediated killing to reduce tumor burden; in addition, CD40L presentation to APCs motivated crosstalk with other effector cells to dampen the pro-angiogenic and immunosuppressive capabilities of tumor-infiltrating immune cells, delaying prostate tumor growth (53). Overall, through CD1d-iNKT TCR recognition, iNKT cells are poised to target TAMs within the solid TME, leading to improved outcomes for cancer patients.

The predominant clinical platforms implementing iNKT immunotherapy utilize autologously engrafted PBMC-derived iNKT cells that are activated prior using *in vitro* glycolipid presentation (54–56). While iNKT-based therapy could be repurposed for TAM depletion, limitations on PBMC iNKT purity and yield demand a novel platform for the generation of iNKT cells. To overcome this hurdle, our group explored *in vitro* generation of allogenic hematopoietic stem cell (HSC)-engineered iNKT (^Allo^HSC-iNKT) cells through cord blood HSC engineering; notably the final cell product displayed near one-thousand-fold expansion, providing a scalable platform for extensive generation of ^Allo^HSC-iNKT cells (48, 50). In agreement with the anti-TAM function of PBMC iNKT cells, significant depletion of M2 macrophages and TAMs by ^Allo^HSC-iNKT cells was similarly observed, validating their efficacy (48, 50). Importantly, these engineered iNKT cells specifically depleted virus-infected monocytes through CD1d recognition, suggesting that virus-infected monocytes or TAMs present greater amounts of stress molecules and glycolipids, enhancing cytotoxic recognition of ^Allo^HSC-iNKT cells using intrinsic NK pathways and iNKT TCR pathways (48). Despite their promise, current ^Allo^HSC-iNKT cell products and other stem cell-derived cell therapies confront certain limitations that preclude implementation. During human stem cell culture, induction of mouse-derived stromal feeder cells (e.g., OP9 and MS5 cells) could potentially increase the risk of mouse cell contamination. The manufacturing process can be improved through replacement with a feeder-free culture system to improve the safety profile of cell products and accelerate the clinical development (43, 57). In addition, the highly inflammatory and cytotoxic function of ^Allo^HSC-iNKT cells in conjunction with their rapid *in vitro* and *in vivo* cell proliferation may induce cytokine release/storm syndrome (CRS) as a side-effect during massive lysis of tumor cells and TAMs. Indeed, CRS is a salient concern for current CAR-T therapies (58). So far, the fast evolution of CAR-T cell therapy has accumulated valuable clinical experiences for CRS management (e.g., anti-IL-6 antibody treatment), that can be adapted for ^Allo^HSC-iNKT cell therapy and other stem cell-based cell therapies. Moreover, a suicide gene “safety switch” (e.g., sr39TK, iCasp9, RQR8) could be incorporated in these cellular products to provide an additional safety control (59, 60).

To further overcome tumor development, the ^Allo^HSC-iNKT platform could adopt CAR-engineered iNKT (CAR-iNKT) mechanisms to enhance dual targeting of TAMs and tumor cells. The ^Allo^HSC-iNKT cell product demonstrated cytotoxicity against multiple tumor cell lines (50); the baseline level of tumor killing mediated by NKG2D and other activating NK receptors (52) could thus be further enhanced using CARs for high-fidelity recognition. Successful preliminary trials incorporating CAR-iNKT therapy have targeted a variety of cancer-specific antigens including CD19 (61), B cell maturation antigen (BCMA) (62), and GD2 (63) for treatment of B cell lymphoma, multiple myeloma, and neuroblastoma, respectively (**Table 2**). Precise elimination of tumor cells through CAR targeting reduces secretion of immunosuppressive cytokines (e.g. IL-10 and TGFβ) that induce TAM polarization and persistence (72), thereby minimizing TAM-related immunosuppression. CAR-iNKT cells are highly potent effector cells that remediate the TME through simultaneous depletion of TAMs and tumors using iNKT TCR/CD1d and CAR recognition, respectively, as well as generalized elimination of both mediated by NK receptors (3, 42, 57, 73). Refinement of the HSC-iNKT platform with CAR-engineering provides a powerful method to address the TME, which remains a significant barrier to the efficacy of cell-based treatments, to produce a powerful alternative for current cancer immunotherapies.

**Table 2:** Preclinical and Clinical Trials of Innate T cell CAR-Based Therapies.

Innate T cell type	Preclinical or clinical	CAR antigen	Other engineering	Targeting cancer	Outcome	Reference or No. NCT
iNKT	Clinical	GD2	IL-15 overexpression	Neuroblastoma		NCT03294954
		CD19	IL-15 overexpression	B cell malignancies		NCT04814004 and NCT03774654
	Preclinical	BCMA	HSC engineering, *B2M* and *CIITA* knockout	Multiple myeloma	Enhanced antitumor capacity, multiple antitumor mechanisms, high safety, and low immunogenicity	(50)
			mRNA electroporation	Multiple myeloma	Improved cytotoxicity	(64)
				Multiple myeloma	CAR- and TCR-mediated cytotoxic activity, and *in vivo* expansion with α-GalCer pulsed DCs	(62)
		CD38		Multiple myeloma	CAR- and TCR-mediated cytotoxic activity, and *in vivo* expansion with α-GalCer pulsed DCs	(62)
		CD19		Lymphoma	Enhanced anti-lymphoma activity and dual CD19 and CD1d targeting	(61)
				Lymphoma	Prolonged *in vivo* persistence and superior therapeutic activities	(65)
				Lymphoma	Potent antitumor effect through direct cytotoxicity and host CD8 T cell cross-priming, and no GvHD risk	(66)
				Lymphoma	Enhanced effector functions by IL-21	(67)
		GD2		Neuroblastoma	Potent *in vivo* antitumor activity, anti-TAM capacity, and no GvHD risk	(68)
			IL-15 overexpression	Neuroblastoma	Enhanced *in vivo* persistence and therapeutic efficacy	(63)
		CSPG4	mRNA electroporation	Melanoma	CAR- and TCR-mediated cytotoxic activity	(64)
MAIT	Preclinical	Mesothelin		Ovarian cancer	Potent antitumor and anti-TAM capacity	(27)
γδT	Clinical	CD19		B cell malignancies		NCT02656147
		CD20		B cell malignancies		NCT04735471
		CD7		CD7^+^ T lymphoma		NCT04702841
		NKG2D ligand		Solid tumors		NCT04107142
	Preclinical	CD19	Electroporation with Sleeping Beauty transposon and transposase	Leukemia	Enhanced antitumor capacity	(69)
				Leukemia	CAR-directed and independent antitumor activity, and enhanced cytotoxicity in the presence of zoledronate	(70)
		NKG2D ligand	mRNA electroporation	NKG2D ligand-positive cancer cells	Targeting multiple solid tumor cell lines *in vitro*	(71)

Further incorporation of immune enhancing genes (e.g., IL-15) and depleting checkpoint molecules (e.g., PD-1 and CTLA-4) in iNKT cell- and other immune cell-based therapy have been explored to expand CAR performance. Self-sustaining secretion of human IL-15 through CAR integration presages activation of essential signaling for iNKT and NK cell development and homeostasis, enhancing *in vivo* persistence and antitumor function (74–77). A GD2-targeting CAR-armed, IL-15-enhanced iNKT cell product was applied to clinical trials for treating children with relapsed neuroblastoma, demonstrating encouraging therapeutic outcomes, safety, and feasibility (63, 78). Blockade of checkpoints such as PD-1 and CTLA-4 significantly enhanced the antitumor immunity of human iNKT and other immune cells (79, 80). In a similar vein, CRIPSR-Cas 9 technology has been successfully used to knock out checkpoints to enhance antitumor immunity in cytotoxic T lymphocytes, and such technology could be easily applied to other cell types (81–83). Considering TAMs engineer immunosuppression through upregulation of PD-1 ligands (e.g., PD-L1 and PD-L2) (3, 84), depleting cognate checkpoint molecules in iNKT cells and other immune cells provides another promising strategy to enhance their anti-TAM capacities and persistence, therefore augmenting tumor treatment.

## Targeting the tumor microenvironment using MAIT cells

Mucosal-associated invariant T (MAIT) cells are another innate-like T cell subset that recognize small molecule biosynthetic derivatives produced during microbial riboflavin synthesis, which are then presented on MHC-related protein-1 (MR1) by APCs (85–88). Mouse MAIT TCR is comprised of a semi-invariant TCR α chain Vα19–Jα33 predominantly paired with TCRβ chain Vβ6/Vβ8; human MAIT TCR uses Vα7.2–Jα33/12/20 associated with Vβ2/Vβ13 (89, 90). MAIT cell development in the thymus derives from a common CD4^+^CD8^+^ DP αβ T cell progenitor as conventional T cells; MR1-mediated positive selection of MAIT TCR by TECs induces differential co-expression of CD161 and CD8αα markers, controlled by PLZF transcription factor (91, 92). Two ligands specifically, 5-(2-oxopropylideneamino)-6-D-ribitylaminouracil (5-OP-RU) and 5-(2-oxoethylideneamino)-6-D-ribitylaminouracil (5-OE-RU), are produced by several strains of bacteria and yeast during riboflavin synthesis; through specific presentation to MAIT TCR these ligands could induce MAIT cell activation, akin to αGC stimulation of iNKT cells (93). Activated MAIT cells expand rapidly and produce an innate-like immune response with potent effector function through secretion of inflammatory cytokines, chemokines, and cytotoxic molecules to eliminate target cells.

Both bone marrow-derived APCs, including monocytes, macrophages, and DCs, and non-bone marrow-derived epithelial cells express high levels of MR1 for MAIT cell activation (94–97). Specifically, elevated expression of MR1 on healthy donor PBMC-derived M2-polarized macrophages and cancer patient endogenous TAMs suggest that MAIT cells could mobilize a powerful anti-TAM response to engineer a TME with pro-inflammatory character (27). Similar to PBMC-derived iNKT cells, MAIT cells could directly target M2 macrophages through intrinsic NK activating receptors; in addition, the application of 5-OP-RU could induce TCR activation and further enhance MAIT cell anti-TAM capacity, which was blocked by the anti-MR1 antibody (**Figure 2C**) (27). Macrophage-killing by MAIT cells was also correlated with upregulation of activation markers, such as CD25, and secretion of pro-inflammatory cytokines, such as IFN-γ (27). Furthermore, MAIT cells could undergo CAR engineering, wherein the mesothelin CAR-armed MAIT (MCAR-MAIT) cells demonstrated dual killing of mesothelin^+^ ovarian tumor cells and MR1^+^ TAMs to enhance antitumor reactivity (27).

## Targeting the tumor microenvironment using γδT cells

Gamma delta T (γδT) cells, another scarce population of unconventional T cells, express rearranged TCR γδ chains instead of conventional TCR αβ chains. Unlike αβT cells, γδT cells possess features of both innate and adaptive immune cells and can be activated in the absence of their cognate TCR ligands through APC cytokine signaling alone (98, 99). γδT cells arise from a common CD4^-^CD8^-^ double negative (DN) progenitor as conventional αβ T cells, but productive rearrangement of γδ TCR rearrangement between DN2 (CD44^+^CD25^+^) and DN3 (CD44^-^CD25^+^) stages induces fate commitment towards the γδ subtype (100). Enrichment through TEC positive selection of γδ TCR induces differential expression of CD73, which is persistently expressed by peripheral γδT cells; CD73 can therefore be used as an early indicator of γδ fate commitment (101, 102). When activated, γδT cells generate a burst of inflammatory cytokines that subsequently induce an inflammatory response from adaptive effector cells (98, 99). These features poise γδT cells as a potent upstream effector T cell that mediates the immune cascade in inflamed tissues (103).

As mentioned previously, γδT cells do not require MHC antigen presentation for recognition and function, evading onset of GvHD observed in allogeneic engraftment of classical αβT cells (104, 105). Amino bisphosphonate class drugs such as zoledronate have been shown to effectively induce γδT cell expansion both *in vitro* and *in vivo* (106, 107). Profiling of *in vitro* expanded Vγ9Vδ2 T cells show potent antitumor functions that hold attractive promise for adoptive immunotherapy (108). Since γδT cells demonstrate intrinsic anti-tumor reactivity and can be safely applied for allogeneic therapies (27), engineering γδT cells with CAR expression provides an off-the-shelf approach to target tumors with higher antigen heterogeneity and lower antigen density, which present an obstacle for conventional CAR-T cells.

Despite their significant antitumor potential, the capacity of γδT cells to modulate the TME remain controversial. Previous studies reported that engraftment of mouse γδT cells induced an undesirable increase in the quantity of MDSCs and mobilized MDSC infiltration, exacerbating the immunosuppressive TME through MDSC-mediated CD8^+^ T cell exhaustion (109, 110). An additional human study related to colorectal cancer revealed that γδT17 cell secretion of IL-17, G-CSF, and GM-CSF cytokine could mobilize polymorphonuclear MDSCs into the tumor, eliciting immunosuppression (111). However, mitigation of γδT cell pro-tumoral effects is achievable through modulation of their cytokine profile to prevent MDSC recruitment; a study of γδ17 in a murine breast cancer model demonstrated the capacity to minimize accumulation and pro-tumoral polarization of neutrophils through ablation of IL-17 and G-CSF pathways (112). In conjunction, an opposing study indicated the strong anti-tumoral capacity of γδT cells through synergistic application of Zoledronate to stimulate cytotoxicity against monocytes, and therefore TAMs, although the γδT cells lacked the capacity to localize to the tumor site (112). We previously utilized an *in vitro* mixed macrophage/γδT cell assay to study the anti-TAM function of γδT cells and verified the killing capacity of allogeneic PBMC-derived γδT against M2 macrophages in the presence of Zoledronate (**Figure 2D**) (27). While elimination of IL-17, G-CSF, and GM-CSF cytokines could potentially be applied to therapeutic γδT cells to reduce their pro-tumoral effects, the impact of such treatment on their anti-tumoral capacity is still unknown (27, 112, 113).

Although the exact mechanism of recognition as well as interplay with MDSC-mediated exhaustion remains under investigation, allogeneic γδT cells could be another promising candidate to target immunosuppressive cells and modulate the TME.

## Discussion

Immunosuppressive cells, especially M2-like TAMs and MDSCs, have been shown to play a role in the progression, metastasis, and chemoresistance of solid tumors (114, 115). Given their role in promoting an immunosuppressive TME in cancer, the specific targeting of TAMs and MDSCs may potentially provide an effective therapeutic route to stimulate patient immune response (116). To date, there have been several proposed methods of targeting M2-like TAMs in cancer *via* various strategies, including the use of immunotherapies, small molecule inhibitors, and nanoparticles (116). The overall goal of these targeted therapies is either outright elimination of TAMs in the TME to prevent further recruitment of TAMs or repolarization of M2-like TAMs towards a pro-inflammatory M1-like phenotype (7, 117).

However, these current treatment strategies are still plagued with their own drawbacks and limitations. The administration of bisphosphonates remains under questionable consideration due to a lack of target specificity within the TAM population; the inability to specifically eliminate M2-like, pro-tumoral TAMs can result in wide-spread TAM depletion that may result in an overactive immune response for potential patients (118). Use of CSF-1R inhibition has also generated inconsistent results, yielding limited clinical and single-agent success (116, 119). Additionally, CSF-1R inhibition for TAM elimination within the TME may undesirably increase MDSC infiltration worsening patient symptomatology (119). For CCL2-directed inhibition therapy, termination of the drug regiment adversely enhanced metastatic progression and decreased survival in mouse breast cancer models (120). Due to the limitations of the current therapies geared towards eliminating TAMs in the TME, a safe, effective alternative is necessary.

Innate T cells, including iNKT, MAIT and γδT cells are unconventional T cell subsets that have the potential to deplete TAMs through powerful NK receptor- and TCR-mediated cytotoxicity (27, 28, 54). These innate-like T cells are activated independently of MHC antigen presentation, and therefore do not recognize mismatch or protein alloantigen to induce GvHD (42, 121–123). The GvHD-free safety profile situate these innate T cell subsets as ideal candidates for the development of an “off-the-shelf” allogeneic cell therapy. Further engineering, such as arming with CARs, incorporating immune enhanced genes (e.g., IL-15), and depleting checkpoint molecules (e.g., PD-1 and CTLA-4), could improve the antitumor immunity of these therapeutic cells and provide an approach to simultaneously target both tumor and immunosuppressive cells. Further enhancement of anti-TAM capacity has been achieved using engineered FRβ CAR-T cells for depletion of FRβ^+^ TAMs (20); incorporation of the aforementioned FRβ CAR on allogeneic innate T cells could achieve powerful anti-TAM killing through an NK/TCR/FRβ CAR triple targeting mechanism.

One of the major limitations for innate T cell-based therapy is their low frequency and number in human. Human blood contains low numbers of iNKT (0.001-1%), MAIT (0.1-5%) and γδT (0.1-5%) cells, making it very difficult to reliably grow large numbers of innate T cells for CAR-engineering (28, 124, 125). Therefore, the initial cell materials require optimized expansion protocols, usually involving agonist (e.g., α-GalCer, 5-OP-RU, and Zoledronate)-loaded feeder cells and cytokines, followed by enrichment, purification and subsequent cell engineering (57). In addition, low viral transduction rate on some innate T cells may limit their CAR engineering and CAR-mediated antitumor functions, and strategies to improve the efficiency of viral transduction on innate T cells can be developed (28, 126).

**Figure 3:**
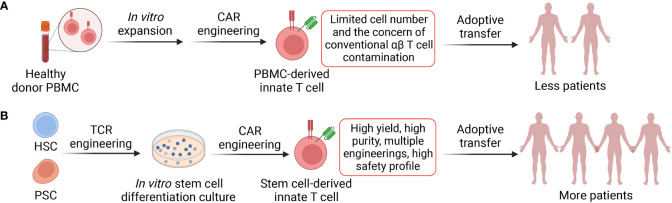
Two approaches to generate CAR-engineered innate T cells. **(A)** Innate T cells are enriched from healthy donor peripheral blood mononuclear cells (PBMCs) *via* cell sorting, cultured *in vitro* and engineered with CARs. The generated CAR-engineered innate T cells could target both tumor and immunosuppressive cells. However, these cells have limited expansion fold and yield; therefore, the cell products could be adoptively transferred to less cancer patients. **(B)** Stem cells such as hematopoietic stem cells (HSCs) and pluripotent stem cells (PSCs; include embryonic stem cells and induced PSCs) could be engineered with innate T cell TCR and then cultured in the *in vitro* differentiation systems where the stem cells develop into mature innate T cells. The platforms could be easily combined with CAR engineering and other genetic modifications such as CRISPR-Cas9. The stem cell-derived innate T cells have high yield and purity and can be transferred to more cancer patients.

Although the scarcity of innate T cells in human peripheral blood hinder the application of these cells, stem cell engineering and *in vitro* differentiation provide another opportunity to generate these cells at high yield and purity (**Figure 3A**) (48, 50). Multiple stem cell sources (e.g., HSCs, ESCs, and iPSCs) and stem cell culture approaches (e.g., OP9-DL, artificial thymic organoid, and Feeder-Free culture) have been employed to generate innate T cells that bear close resemblance to healthy donor PBMC-derived immune cells and maintain their potent tumor targeting capabilities (**Figure 3B**) (48, 50, 127, 128). For example, the OP9-DL system, which is based on a mouse stromal cell line OP9 overexpressing the Notch ligand, Delta-like ligand 1 (DLL-1) or 4 (DLL-4), was utilized to generate iPSC-derived iNKT and MAIT cells; the artificial thymic organoid (ATO) culture system, which is based on DLL-1- or DLL-4- overexpressed mouse stromal cell line MS5, was used to develop HSC-engineered iNKT cells; feeder-free, serum-free culture system has also been developed recently to generate iNKT cells with high yield, purity, and safety profile (43, 48, 50, 127–130). Overall, *in vitro* generation of CAR-engineered innate iNKT, MAIT, and γδT cells have the potential to effectively target both tumor cells and immunosuppressive cells, thus highlighting the capacity of innate T cell-based therapy for treatment of solid tumors, especially in the absence of inflammatory signaling, a defect characteristic of TME afflicted “cold” tumors.

## Author contributions

This manuscript was written by Y-RL, MW, and LY. All authors contributed to the article and approved the submitted version.

## Funding

This work was supported by a Partnering Opportunity for Translational Research Projects Award and a Partnering Opportunity for Discovery Stage Award from the California Institute for Regenerative Medicine (CIRM TRAN1-12250 and DISC2-13505, to LY), a UCLA BSCRC Innovation Award (to LY), and an Ablon Scholars Award (to LY).

## Acknowledgments

The authors would like to acknowledge the members of the Yang Lab for contributing insights and discussion surrounding the topic. Figures were created in biorender.com.

## Conflict of interest

Y-RL and LY are inventors on patents relating to this article filed by UCLA. LY is a scientific advisor to AlzChem and Amberstone Biosciences, and a co-founder, stockholder, and advisory board member of Appia Bio. None of the declared companies contributed to or directed any of the research reported in this article.

The remaining authors declare that the research was conducted in the absence of any commercial or financial relationships that could be construed as a potential conflict of interest.

## Publisher’s note

All claims expressed in this article are solely those of the authors and do not necessarily represent those of their affiliated organizations, or those of the publisher, the editors and the reviewers. Any product that may be evaluated in this article, or claim that may be made by its manufacturer, is not guaranteed or endorsed by the publisher.

## Abbreviations

TME, tumor microenvironment; TAM, tumor-associated macrophage; MDSC, myeloid-derived suppressor cell; iNKT, invariant natural killer T; MAIT, mucosal-associated invariant T; γδT, gamma delta T; CAR, chimeric antigen receptor; GvHD, graft versus host disease; CAF, cancer-associated fibroblasts; PD-L1, programmed death-ligand 1; TGF-β, transforming growth factor-β; TNF-α, tumor necrosis factor-α; VEGF, vascular endothelial growth factor; bFGF, basic fibroblast growth factor; CSF1, colony stimulating factor 1; G-CSF, Granulocyte colony stimulating factor; GM-CSF, Granulocyte macrophage colony stimulating factor; CRS, cytokine release/storm syndrome; DC, dendritic cell; APC, antigen-presenting cell; MR1, MHC-related protein-1; HSC, hematopoietic stem cell; ESC, embryonic stem cell; iPSC, induced pluripotent stem cell; ATO, artificial thymic organoid.

## References

[1] Wu T Dai Y. Tumor microenvironment and therapeutic response. Cancer Lett (2017) 387:61–8. doi: 10.1016/j.canlet.2016.01.043 26845449

[2] Denton AE Roberts EW Fearon DT. Stromal cells in the tumor microenvironment. Adv Exp Med Biol (2018) 1060:99–114. doi: 10.1007/978-3-319-78127-3_6 30155624

[3] Li Y-R Yu Y Kramer A Hon R Wilson M Brown J. An ex vivo 3D tumor microenvironment-mimicry culture to study TAM modulation of cancer immunotherapy. Cells (2022) 11:1583. doi: 10.3390/cells11091583 35563889 PMC9101510

[4] Caux C Ramos RN Prendergast GC Bendriss-Vermare N Ménétrier-Caux C. A milestone review on how macrophages affect tumor growth. Cancer Res (2016) 76:6439–42. doi: 10.1158/0008-5472.CAN-16-2631 28148676

[5] Donnem T Hu J Ferguson M Adighibe O Snell C Harris AL. Vessel co-option in primary human tumors and metastases: An obstacle to effective anti-angiogenic treatment? Cancer Med (2013) 2:427–36. doi: 10.1002/cam4.105 PMC3799277 24156015

[6] Fei L Ren X Yu H Zhan Y. Targeting the CCL2/CCR2 axis in cancer immunotherapy: One stone, three birds? Front Immunol (2021) 12:771210. doi: 10.3389/fimmu.2021.771210 34804061 PMC8596464

[7] Petty AJ Owen DH Yang Y Huang X. Targeting tumor-associated macrophages in cancer immunotherapy. Cancers (Basel) (2021) 13:5318. doi: 10.3390/cancers13215318 34771482 PMC8582510

[8] Li M He L Zhu J Zhang P Liang S. Targeting tumor-associated macrophages for cancer treatment. Cell Biosci (2022) 12:85. doi: 10.1186/s13578-022-00823-5 35672862 PMC9172100

[9] Lee C Jeong H Bae Y Shin K Kang S Kim H. Targeting of M2-like tumor-associated macrophages with a melittin-based pro-apoptotic peptide. J Immunother Cancer (2019) 7:147. doi: 10.1186/s40425-019-0610-4 31174610 PMC6555931

[10] Opperman KS Vandyke K Clark KC Coulter EA Hewett DR Mrozik KM. Clodronate-liposome mediated macrophage depletion abrogates multiple myeloma tumor establishment *In vivo* . Neoplasia (2019) 21:777–87. doi: 10.1016/j.neo.2019.05.006 PMC6593350 31247457

[11] Li Y Wu H Ji B Qian W Xia S Wang L. Targeted imaging of CD206 expressing tumor-associated M2-like macrophages using mannose-conjugated antibiofouling magnetic iron oxide nanoparticles. ACS Appl Bio Mater (2020) 3:4335–47. doi: 10.1021/acsabm.0c00368 PMC8626204 34841220

[12] Zhang F Parayath NN Ene CI Stephan SB Koehne AL Coon ME. Genetic programming of macrophages to perform anti-tumor functions using targeted mRNA nanocarriers. Nat Commun (2019) 10:3974. doi: 10.1038/s41467-019-11911-5 31481662 PMC6722139

[13] Andersen MN Etzerodt A Graversen JH Holthof LC Moestrup SK Hokland M. STAT3 inhibition specifically in human monocytes and macrophages by CD163-targeted corosolic acid-containing liposomes. Cancer Immunol Immunother (2019) 68:489–502. doi: 10.1007/s00262-019-02301-3 30637473 PMC11028169

[14] Wanderley CW Colón DF Luiz JPM Oliveira FF Viacava PR Leite CA. Paclitaxel reduces tumor growth by reprogramming tumor-associated macrophages to an M1 profile in a TLR4-dependent manner. Cancer Res (2018) 78:5891–900. doi: 10.1158/0008-5472.CAN-17-3480 30104241

[15] Tan H-Y Wang N Man K Tsao S-W Che C-M Feng Y. Autophagy-induced RelB/p52 activation mediates tumour-associated macrophage repolarisation and suppression of hepatocellular carcinoma by natural compound baicalin. Cell Death Dis (2015) 6:e1942. doi: 10.1038/cddis.2015.271 26492375 PMC4632300

[16] Buhtoiarov IN Sondel PM Wigginton JM Buhtoiarova TN Yanke EM Mahvi DA. Anti-tumour synergy of cytotoxic chemotherapy and anti-CD40 plus CpG-ODN immunotherapy through repolarization of tumour-associated macrophages. Immunology (2011) 132:226–39. doi: 10.1111/j.1365-2567.2010.03357.x PMC3050446 21039467

[17] Di Caro G Cortese N Castino GF Grizzi F Gavazzi F Ridolfi C. Dual prognostic significance of tumour-associated macrophages in human pancreatic adenocarcinoma treated or untreated with chemotherapy. Gut (2016) 65:1710–20. doi: 10.1136/gutjnl-2015-309193 26156960

[18] Rodriguez-Garcia A Palazon A Noguera-Ortega E Powell DJ Jr. Guedan S. CAR-T cells hit the tumor microenvironment: Strategies to overcome tumor escape. Front Immunol (2020) 11:1109. doi: 10.3389/fimmu.2020.01109 32625204 PMC7311654

[19] Ruella M Kenderian SS. Next-generation chimeric antigen receptor T-cell therapy: Going off the shelf. BioDrugs (2017) 31:473–81. doi: 10.1007/s40259-017-0247-0 PMC5699508 29143249

[20] Rodriguez-Garcia A Lynn RC Poussin M Eiva MA Shaw LC O’Connor RS. CAR-T cell-mediated depletion of immunosuppressive tumor-associated macrophages promotes endogenous antitumor immunity and augments adoptive immunotherapy. Nat Commun (2021) 12:877. doi: 10.1038/s41467-021-20893-2 33563975 PMC7873057

[21] Bughda R Dimou P D’Souza RR Klampatsa A. Fibroblast activation protein (FAP)-targeted CAR-T cells: Launching an attack on tumor stroma. ImmunoTargets Ther (2021) 10:313–23. doi: 10.2147/ITT.S291767 PMC8354246 34386436

[22] Petrausch U Schuberth PC Hagedorn C Soltermann A Tomaszek S Stahel R. Re-directed T cells for the treatment of fibroblast activation protein (FAP)-positive malignant pleural mesothelioma (FAPME-1). BMC Cancer (2012) 12:615. doi: 10.1186/1471-2407-12-615 23259649 PMC3585825

[23] Uckun FM Watts J. CD123-directed bispecific antibodies for targeting MDS clones and immunosuppressive myeloid-derived suppressor cells (MDSC) in high-risk adult MDS patients. Front Aging (2021) 2:757276. doi: 10.3389/fragi.2021.757276 35822053 PMC9261311

[24] Gustafson M Lin Y Maas M Gastineau D Dietz A. A method for non-overlapping identification of human myeloid derived suppressor cells. J Immunother Cancer (2014) 2:P150. doi: 10.1186/2051-1426-2-S3-P150

[25] Perera LP Zhang M Nakagawa M Petrus MN Maeda M Kadin ME. Chimeric antigen receptor modified T cells that target chemokine receptor CCR4 as a therapeutic modality for T-cell malignancies. Am J Hematol (2017) 92:892–901. doi: 10.1002/ajh.24794 28543380 PMC5546946

[26] Nicolay JP Albrecht JD Alberti-Violetti S Berti E. CCR4 in cutaneous T-cell lymphoma: Therapeutic targeting of a pathogenic driver. Eur J Immunol (2021) 51:1660–71. doi: 10.1002/eji.202049043 33811642

[27] Li Y-R Brown J Yu Y Lee D Zhou K Dunn ZS. Targeting immunosuppressive tumor-associated macrophages using innate T cells for enhanced antitumor reactivity. Cancers (2022) 14:2749. doi: 10.3390/cancers14112749 35681730 PMC9179365

[28] Cortés-Selva D Dasgupta B Singh S Grewal IS. Innate and innate-like cells: The future of chimeric antigen receptor (CAR) cell therapy. Trends Pharmacol Sci (2021) 42:45–59. doi: 10.1016/j.tips.2020.11.004 33250273

[29] Van Kaer L Parekh VV Wu L. Invariant natural killer T cells: Bridging innate and adaptive immunity. Cell Tissue Res (2011) 343:43–55. doi: 10.1007/s00441-010-1023-3 20734065 PMC3616393

[30] Zhou Y Li Y-R Zeng S Yang L. Methods for studying mouse and human invariant natural killer T cells. Methods Mol Biol (2021) 2388:35–57. doi: 10.1007/978-1-0716-1775-5_4 34524660

[31] Juno JA Keynan Y Fowke KR. Invariant NKT cells: Regulation and function during viral infection. PloS Pathog (2012) 8:e1002838. doi: 10.1371/journal.ppat.1002838 22916008 PMC3420949

[32] Nelson A Lukacs JD Johnston B. The current landscape of NKT cell immunotherapy and the hills ahead. Cancers (2021) 13:5174. doi: 10.3390/cancers13205174 34680322 PMC8533824

[33] Park SH Weiss A Benlagha K Kyin T Teyton L Bendelac A. The mouse CD1d-restricted repertoire is dominated by a few autoreactive T cell receptor families. J Exp Med (2001) 193:893–904. doi: 10.1084/jem.193.8.893 11304550 PMC2193401

[34] Cardell S Tangri S Chan S Kronenberg M Benoist C Mathis D. CD1-restricted CD4+ T cells in major histocompatibility complex class II-deficient mice. J Exp Med (1995) 182:993–1004. doi: 10.1084/jem.182.4.993 7561702 PMC2192275

[35] Kawano T Cui J Koezuka Y Toura I Kaneko Y Motoki K. CD1d-restricted and TCR-mediated activation of valpha14 NKT cells by glycosylceramides. Science (1997) 278:1626–9. doi: 10.1126/science.278.5343.1626 9374463

[36] Brossay L Chioda M Burdin N Koezuka Y Casorati G Dellabona P. CD1d-mediated recognition of an alpha-galactosylceramide by natural killer T cells is highly conserved through mammalian evolution. J Exp Med (1998) 188:1521–8. doi: 10.1084/jem.188.8.1521 PMC2213408 9782129

[37] Gapin L Matsuda JL Surh CD Kronenberg M. NKT cells derive from double-positive thymocytes that are positively selected by CD1d. Nat Immunol (2001) 2:971–8. doi: 10.1038/ni710 11550008

[38] Baranek T de Amat Herbozo C Mallevaey T Paget C. Deconstructing iNKT cell development at single-cell resolution. Trends Immunol (2022) 43:503–12. doi: 10.1016/j.it.2022.04.012 35654639

[39] Nagarajan NA Kronenberg M. Invariant NKT cells amplify the innate immune response to lipopolysaccharide. J Immunol (2007) 178:2706–13. doi: 10.4049/jimmunol.178.5.2706 17312112

[40] Reilly EC Wands JR Brossay L. Cytokine dependent and independent iNKT cell activation. Cytokine (2010) 51:227–31. doi: 10.1016/j.cyto.2010.04.016 PMC2914806 20554220

[41] Zhu Y Smith DJ Zhou Y Li YR Yu J Lee D. Development of hematopoietic stem cell-engineered invariant natural killer T cell therapy for cancer. Cell Stem Cell (2019) 25:542–557.e9. doi: 10.1016/j.stem.2019.08.004 31495780 PMC7018522

[42] Li Y-R Dunn ZS Zhou Y Lee D Yang L. Development of stem cell-derived immune cells for off-the-Shelf cancer immunotherapies. Cells (2021) 10:3497. doi: 10.3390/cells10123497 34944002 PMC8700013

[43] Li Y-R Zeng S Dunn ZS Zhou Y Li Z Yu J. Off-the-shelf third-party HSC-engineered iNKT cells for ameliorating GvHD while preserving GvL effect in the treatment of blood cancers. iScience (2022) 25:104859. doi: 10.1016/j.isci.2022.104859 36034226 PMC9399487

[44] Metelitsa LS. Anti-tumor potential of type-I NKT cells against CD1d-positive and CD1d-negative tumors in humans. Clin Immunol (2011) 140:119–29. doi: 10.1016/j.clim.2010.10.005 PMC3444285 21095162

[45] Song L Asgharzadeh S Salo J Engell K Wu H Sposto R. Vα24-invariant NKT cells mediate antitumor activity *via* killing of tumor-associated macrophages. J Clin Invest (2009) 119:1524–36. doi: 10.1172/JCI37869 PMC2689106 19411762

[46] Lanier LL. NKG2D receptor and its ligands in host defense. Cancer Immunol Res (2015) 3:575–82. doi: 10.1158/2326-6066.CIR-15-0098 PMC4457299 26041808

[47] An B Lim J-Y Jeong S Shin D-M Choi EY Min C-K. CD1d is a novel cell-surface marker for human monocytic myeloid-derived suppressor cells with T cell suppression activity in peripheral blood after allogeneic hematopoietic stem cell transplantation. Biochem Biophys Res Commun (2018) 495:519–25. doi: 10.1016/j.bbrc.2017.11.010 29108995

[48] Li YR Dunn ZS Garcia G Jr. Carmona C Zhou Y Lee D. Development of off − the − shelf hematopoietic stem cell − engineered invariant natural killer T cells for COVID − 19 therapeutic intervention. Stem Cell Res Ther (2022) 13:1–15. doi: 10.1186/s13287-022-02787-2 35313965 PMC8935266

[49] Aoki T Takami M Takatani T Motoyoshi K Ishii A Hara A. Activated invariant natural killer T cells directly recognize leukemia cells in a CD1d-independent manner. Cancer Sci (2020) 111:2223–33. doi: 10.1111/cas.14428 PMC7385353 32324315

[50] Li Y-R Zhou Y Kim YJ Zhu Y Ma F Yu J. Development of allogeneic HSC-engineered iNKT cells for off-the-shelf cancer immunotherapy. Cell Rep Med (2021) 2:100449. doi: 10.1016/j.xcrm.2021.100449 34841295 PMC8607011

[51] Krijgsman D Hokland M Kuppen PJK. The role of natural killer T cells in cancer-a phenotypical and functional approach. Front Immunol (2018) 9:367. doi: 10.3389/fimmu.2018.00367 29535734 PMC5835336

[52] Fuertes MB Domaica CI Zwirner NW. Leveraging NKG2D ligands in immuno-oncology. Front Immunol (2021) 12:713158. doi: 10.3389/fimmu.2021.713158 34394116 PMC8358801

[53] Cortesi F Delfanti G Grilli A Calcinotto A Gorini F Pucci F. Bimodal CD40/Fas-dependent crosstalk between iNKT cells and tumor-associated macrophages impairs prostate cancer progression. Cell Rep (2018) 22:3006–20. doi: 10.1016/j.celrep.2018.02.058 29539427

[54] Delfanti G Dellabona P Casorati G Fedeli M. Adoptive immunotherapy with engineered iNKT cells to target cancer cells and the suppressive microenvironment. Front Med (2022) 9:897750. doi: 10.3389/fmed.2022.897750 PMC9125179 35615083

[55] Gao Y Guo J Bao X Xiong F Ma Y Tan B. Adoptive transfer of autologous invariant natural killer T cells as immunotherapy for advanced hepatocellular carcinoma: A phase I clinical trial. Oncologist (2021) 26:e1919–30. doi: 10.1002/onco.13899 PMC8571770 34255901

[56] Cheng X Wang J Qiu C Jin Y Xia B Qin R. Feasibility of iNKT cell and PD-1+CD8+ T cell-based immunotherapy in patients with lung adenocarcinoma: Preliminary results of a phase I/II clinical trial. Clin Immunol (2022) 238:108992. doi: 10.1016/j.clim.2022.108992 35367396

[57] Li Y-R Zhou Y Kramer A Yang L. Engineering stem cells for cancer immunotherapy. Trends Cancer (2021) 7:1059–73. doi: 10.1016/j.trecan.2021.08.004 34479851

[58] Brudno JN Kochenderfer JN. Toxicities of chimeric antigen receptor T cells: recognition and management. Blood (2016) 127:3321–30. doi: 10.1182/blood-2016-04-703751 PMC4929924 27207799

[59] Di Stasi A Tey S-K Dotti G Fujita Y Kennedy-Nasser A Martinez C. Inducible apoptosis as a safety switch for adoptive cell therapy. N Engl J Med (2011) 365:1673–83. doi: 10.1056/nejmoa1106152 PMC3236370 22047558

[60] Straathof KC Pulè MA Yotnda P Dotti G Vanin EF Brenner MK. An inducible caspase 9 safety switch for T-cell therapy. Blood (2005) 105:4247–54. doi: 10.1182/blood-2004-11-4564 PMC1895037 15728125

[61] Rotolo A Caputo VS Holubova M Baxan N Dubois O Chaudhry MS. Enhanced anti-lymphoma activity of CAR19-iNKT cells underpinned by dual CD19 and CD1d targeting. Cancer Cell (2018) 34:596–610.e11. doi: 10.1016/j.ccell.2018.08.017 30300581 PMC6179961

[62] Poels R Drent E Lameris R Katsarou A Themeli M van der Vliet HJ. Preclinical evaluation of invariant natural killer T cells modified with CD38 or BCMA chimeric antigen receptors for multiple myeloma. Int J Mol Sci (2021) 22:1096. doi: 10.3390/ijms22031096 33499253 PMC7865760

[63] Xu X Huang W Heczey A Liu D Guo L Wood M. NKT cells coexpressing a GD2-specific chimeric antigen receptor and IL15 show enhanced *in vivo* persistence and antitumor activity against neuroblastoma. Clin Cancer Res (2019) 25:7126–38. doi: 10.1158/1078-0432.CCR-19-0421 PMC6891170 31484667

[64] Simon B Wiesinger M März J Wistuba-Hamprecht K Weide B Schuler-Thurner B. The generation of CAR-transfected natural killer T cells for the immunotherapy of melanoma. Int J Mol Sci (2018) 19:2365. doi: 10.3390/ijms19082365 PMC6121949 30103488

[65] Tian G Courtney AN Jena B Heczey A Liu D Marinova E. CD62L+ NKT cells have prolonged persistence and antitumor activity *in vivo* . J Clin Invest (2016) 126:2341–55. doi: 10.1172/JCI83476 PMC4887157 27183388

[66] Simonetta F Lohmeyer JK Hirai T Maas-Bauer K Alvarez M Wenokur AS. Allogeneic CAR invariant natural killer T cells exert potent antitumor effects through host CD8 T-cell cross-priming. Clin Cancer Res an Off J Am Assoc Cancer Res (2021) 27:6054–64. doi: 10.1158/1078-0432.CCR-21-1329 PMC8563377 34376537

[67] Ngai H Tian G Courtney AN Ravari SB Guo L Liu B. IL-21 selectively protects CD62L(+) NKT cells and enhances their effector functions for adoptive immunotherapy. J Immunol (2018) 201:2141–53. doi: 10.4049/jimmunol.1800429 PMC6143411 30111631

[68] Heczey A Liu D Tian G Courtney AN Wei J Marinova E. Invariant NKT cells with chimeric antigen receptor provide a novel platform for safe and effective cancer immunotherapy. Blood (2014) 124:2824–33. doi: 10.1182/blood-2013-11-541235 PMC4215313 25049283

[69] Deniger DC Switzer K Mi T Maiti S Hurton L Singh H. Bispecific T-cells expressing polyclonal repertoire of endogenous γδ T-cell receptors and introduced CD19-specific chimeric antigen receptor. Mol Ther (2013) 21:638–47. doi: 10.1038/mt.2012.267 PMC3589159 23295945

[70] Rozenbaum M Meir A Aharony Y Itzhaki O Schachter J Bank I. Gamma-delta CAR-T cells show CAR-directed and independent activity against leukemia. Front Immunol (2020) 11:1347. doi: 10.3389/fimmu.2020.01347 32714329 PMC7343910

[71] Ang WX Ng YY Xiao L Chen C Li Z Chi Z. Electroporation of NKG2D RNA CAR improves Vγ9Vδ2 T cell responses against human solid tumor xenografts. Mol Ther Oncol (2020) 17:421–30. doi: 10.1016/j.omto.2020.04.013 PMC7240063 32462079

[72] Sica A Larghi P Mancino A Rubino L Porta C Totaro MG. Macrophage polarization in tumour progression. Semin Cancer Biol (2008) 18:349–55. doi: 10.1016/j.semcancer.2008.03.004 18467122

[73] Li Y-R Zhou Y Wilson M Kramer A Hon R Zhu Y. Tumor-localized administration of &alpha;-GalCer to recruit invariant natural killer T cells and enhance their antitumor activity against solid tumors. Int J Mol Sci (2022) 23:7547. doi: 10.3390/ijms23147547 35886891 PMC9317565

[74] Liu E Marin D Banerjee P Macapinlac HA Thompson P Basar R. Use of CAR-transduced natural killer cells in CD19-positive lymphoid tumors. N Engl J Med (2020) 382:545–53. doi: 10.1056/nejmoa1910607 PMC7101242 32023374

[75] Christodoulou I Ho WJ Marple A Ravich JW Tam A Rahnama R. Engineering CAR-NK cells to secrete IL-15 sustains their anti-AML functionality but is associated with systemic toxicities. J Immunother Cancer (2021) 9:e003894. doi: 10.1136/jitc-2021-003894 34896980 PMC8655609

[76] Christodoulou I Koldobskiy M Ho WJ Marple A Ravich WJ Rahnama R. Engineered interleukin-15 autocrine signaling invigorates anti-CD123 CAR-NK cells. Blood (2021) 138:2806. doi: 10.1182/blood-2021-146609

[77] Du Z Ng YY Zha S Wang S. piggyBac system to co-express NKG2D CAR and IL-15 to augment the *in vivo* persistence and anti-AML activity of human peripheral blood NK cells. Mol Ther - Methods Clin Dev (2021) 23:582–96. doi: 10.1016/j.omtm.2021.10.014 PMC8609108 34853803

[78] Heczey A Courtney AN Montalbano A Robinson S Liu K Li M. Anti-GD2 CAR-NKT cells in patients with relapsed or refractory neuroblastoma: An interim analysis. Nat Med (2020) 26:1686–90. doi: 10.1038/s41591-020-1074-2 33046868

[79] Kamata T Suzuki A Mise N Ihara F Takami M Makita Y. Blockade of programmed death-1/programmed death ligand pathway enhances the antitumor immunity of human invariant natural killer T cells. Cancer Immunol Immunother (2016) 65:1477–89. doi: 10.1007/s00262-016-1901-y PMC5099366 27631416

[80] Catafal-Tardos E Baglioni MV Bekiaris V. Inhibiting the unconventionals: Importance of immune checkpoint receptors in γδ t, mait, and nkt cells. Cancers (Basel) (2021) 13:4647. doi: 10.3390/cancers13184647 34572874 PMC8467786

[81] Zhao Z Shi L Zhang W Han J Zhang S Fu Z. CRISPR knock out of programmed cell death protein 1 enhances anti-tumor activity of cytotoxic T lymphocytes. Oncotarget (2018) 9:5208–15. doi: 10.18632/oncotarget.23730 PMC5797044 29435173

[82] McGowan E Lin Q Ma G Yin H Chen S Lin Y. PD-1 disrupted CAR-T cells in the treatment of solid tumors: Promises and challenges. BioMed Pharmacother (2020) 121:109625. doi: 10.1016/j.biopha.2019.109625 31733578

[83] Shi L Meng T Zhao Z Han J Zhang W Gao F. CRISPR knock out CTLA-4 enhances the anti-tumor activity of cytotoxic T lymphocytes. Gene (2017) 636:36–41. doi: 10.1016/j.gene.2017.09.010 28888577

[84] Lin Y Xu J Lan H. Tumor-associated macrophages in tumor metastasis: Biological roles and clinical therapeutic applications. J Hematol Oncol (2019) 12:76. doi: 10.1186/s13045-019-0760-3 31300030 PMC6626377

[85] Eckle SBG Corbett AJ Keller AN Chen Z Godfrey DI Liu L. Recognition of vitamin b precursors and byproducts by mucosal associated invariant T cells. J Biol Chem (2015) 290:30204–11. doi: 10.1074/jbc.R115.685990 PMC4683245 26468291

[86] Gao Y Williams AP. Role of innate T cells in anti-bacterial immunity. Front Immunol (2015) 6:302. doi: 10.3389/fimmu.2015.00302 26124758 PMC4463001

[87] Godfrey DI Uldrich AP McCluskey J Rossjohn J Moody DB. The burgeoning family of unconventional T cells. Nat Immunol (2015) 16:1114–23. doi: 10.1038/ni.3298 26482978

[88] Lamichhane R Ussher JE. Expression and trafficking of MR1. Immunology (2017) 151:270–9. doi: 10.1111/imm.12744 PMC5461101 28419492

[89] Reantragoon R Corbett AJ Sakala IG Gherardin NA Furness JB Chen Z. Antigen-loaded MR1 tetramers define T cell receptor heterogeneity in mucosal-associated invariant T cells. J Exp Med (2013) 210:2305–20. doi: 10.1084/jem.20130958 PMC3804952 24101382

[90] Lepore M Kalinichenko A Colone A Paleja B Singhal A Tschumi A. Parallel T-cell cloning and deep sequencing of human MAIT cells reveal stable oligoclonal TCRβ repertoire. Nat Commun (2014) 5:3866. doi: 10.1038/ncomms4866 24832684

[91] Seach N Guerri L Le Bourhis L Mburu Y Cui Y Bessoles S. Double-positive thymocytes select mucosal-associated invariant T cells. J Immunol (2013) 191:6002–9. doi: 10.4049/jimmunol.1301212 24244014

[92] Koay H-F Gherardin NA Enders A Loh L Mackay LK Almeida CF. A three-stage intrathymic development pathway for the mucosal-associated invariant T cell lineage. Nat Immunol (2016) 17:1300–11. doi: 10.1038/ni.3565 27668799

[93] Hinks TSC Zhang X-W. MAIT cell activation and functions. Front Immunol (2020) 11:1014. doi: 10.3389/fimmu.2020.01014 32536923 PMC7267072

[94] van Wilgenburg B Scherwitzl I Hutchinson EC Leng T Kurioka A Kulicke C. MAIT cells are activated during human viral infections. Nat Commun (2016) 7:11653. doi: 10.1038/ncomms11653 27337592 PMC4931007

[95] Salerno-Goncalves R Rezwan T Sztein M. B cells modulate mucosal associated invariant T cell immune responses. Front Immunol (2014) 4:511. doi: 10.3389/fimmu.2013.00511 24432025 PMC3882667

[96] Huimeng W Lars K-N Mai S Criselle D Pediongco TJ Hanwei C. IL-23 costimulates antigen-specific MAIT cell activation and enables vaccination against bacterial infection. Sci Immunol (2019) 4:eaaw0402. doi: 10.1126/sciimmunol.aaw0402 31732518

[97] Dusseaux M Martin E Serriari N Péguillet I Premel V Louis D. Human MAIT cells are xenobiotic-resistant, tissue-targeted, CD161hi IL-17–secreting T cells. Blood (2011) 117:1250–9. doi: 10.1182/blood-2010-08-303339 21084709

[98] Born WK Jin N Aydintug MK Wands JM French JD Roark CL. γδ T lymphocytes–selectable cells within the innate system? J Clin Immunol (2007) 27:133–44. doi: 10.1007/s10875-007-9077-z 17333410

[99] Lalor SJ Dungan LS Sutton CE Basdeo SA Fletcher JM Mills KHG. Caspase-1–processed cytokines IL-1β and IL-18 promote IL-17 production by γδ and CD4 T cells that mediate autoimmunity. J Immunol (2011) 186:5738 LP – 5748. doi: 10.4049/jimmunol.1003597 21471445

[100] Passoni L Hoffman ES Kim S Crompton T Pao W Dong MQ. Intrathymic delta selection events in gammadelta cell development. Immunity (1997) 7:83–95. doi: 10.1016/s1074-7613(00)80512-9 9252122

[101] Coffey F Lee S-Y Buus TB Lauritsen J-PH Wong GW Joachims ML. The TCR ligand-inducible expression of CD73 marks γδ lineage commitment and a metastable intermediate in effector specification. J Exp Med (2014) 211:329–43. doi: 10.1084/jem.20131540 PMC3920555 24493796

[102] Fahl SP Coffey F Wiest DL. Origins of γδ T cell effector subsets: a riddle wrapped in an enigma. J Immunol (2014) 193:4289–94. doi: 10.4049/jimmunol.1401813 25326547

[103] Malik S Want MY Awasthi A. The emerging roles of gamma–delta T cells in tissue inflammation in experimental autoimmune encephalomyelitis. Front Immunol (2016) 7:14. doi: 10.3389/fimmu.2016.00014 26858718 PMC4731487

[104] Legut M Cole DK Sewell AK. The promise of γδ T cells and the γδ T cell receptor for cancer immunotherapy. Cell Mol Immunol (2015) 12:656–68. doi: 10.1038/cmi.2015.28 PMC4716630 25864915

[105] Airoldi I Bertaina A Prigione I Zorzoli A Pagliara D Cocco C. γδ T-cell reconstitution after HLA-haploidentical hematopoietic transplantation depleted of TCR-αβ+/CD19+ lymphocytes. Blood (2015) 125:2349–58. doi: 10.1182/blood-2014-09-599423 PMC4440890 25612623

[106] Fournié J-J Sicard H Poupot M Bezombes C Blanc A Romagné F. What lessons can be learned from γδ T cell-based cancer immunotherapy trials? Cell Mol Immunol (2013) 10:35–41. doi: 10.1038/cmi.2012.39 23241899 PMC4003170

[107] Sugie T Murata-Hirai K Iwasaki M Morita CT Li W Okamura H. Zoledronic acid-induced expansion of γδ T cells from early-stage breast cancer patients: Effect of IL-18 on helper NK cells. Cancer Immunol Immunother (2013) 62:677—87. doi: 10.1007/s00262-012-1368-4 23151944 PMC3639312

[108] Deniger DC Moyes JS Cooper LJN. Clinical applications of gamma delta T cells with multivalent immunity. Front Immunol (2014) 5:636. doi: 10.3389/fimmu.2014.00636 25566249 PMC4263175

[109] Kong X Sun R Chen Y Wei H Tian Z. γδT cells drive myeloid-derived suppressor cell-mediated CD8+ T cell exhaustion in hepatitis b virus-induced immunotolerance. J Immunol (2014) 193:1645–53. doi: 10.4049/jimmunol.1303432 25015833

[110] Qu P Wang L-Z Lin PC. Expansion and functions of myeloid-derived suppressor cells in the tumor microenvironment. Cancer Lett (2016) 380:253–6. doi: 10.1016/j.canlet.2015.10.022 PMC7477794 26519756

[111] Wu P Wu D Ni C Ye J Chen W Hu G. γδT17 cells promote the accumulation and expansion of myeloid-derived suppressor cells in human colorectal cancer. Immunity (2014) 40:785–800. doi: 10.1016/j.immuni.2014.03.013 24816404 PMC4716654

[112] Fowler DW Copier J Dalgleish AG Bodman-Smith MD. Zoledronic acid causes γδ T cells to target monocytes and down-modulate inflammatory homing. Immunology (2014) 143:539–49. doi: 10.1111/imm.12331 PMC4253502 24912747

[113] Coffelt SB Kersten K Doornebal CW Weiden J Vrijland K Hau C-S. IL-17-producing γδ T cells and neutrophils conspire to promote breast cancer metastasis. Nature (2015) 522:345–8. doi: 10.1038/nature14282 PMC4475637 25822788

[114] Cheng H Wang Z Fu L Xu T. Macrophage polarization in the development and progression of ovarian cancers: An overview. Front Oncol (2019) 9:421. doi: 10.3389/fonc.2019.00421 31192126 PMC6540821

[115] Nowak M Klink M. The role of tumor-associated macrophages in the progression and chemoresistance of ovarian cancer. Cells (2020) 9:1299. doi: 10.3390/cells9051299 PMC7290435 32456078

[116] Guerriero JL. Macrophages: The road less traveled, changing anticancer therapy. Trends Mol Med (2018) 24:472–89. doi: 10.1016/j.molmed.2018.03.006 PMC5927840 29655673

[117] Li C Xu X Wei S Jiang P Xue L Wang J. Tumor-associated macrophages: potential therapeutic strategies and future prospects in cancer. J Immunother Cancer (2021) 9:e001341. doi: 10.1136/jitc-2020-001341 33504575 PMC8728363

[118] Xiang X Wang J Lu D Xu X. Targeting tumor-associated macrophages to synergize tumor immunotherapy. Signal Transduct Target Ther (2021) 6:75. doi: 10.1038/s41392-021-00484-9 33619259 PMC7900181

[119] Kumar V Donthireddy L Marvel D Condamine T Wang F Lavilla-Alonso S. Cancer-associated fibroblasts neutralize the anti-tumor effect of CSF1 receptor blockade by inducing PMN-MDSC infiltration of tumors. Cancer Cell (2017) 32:654–668.e5. doi: 10.1016/j.ccell.2017.10.005 29136508 PMC5827952

[120] Bonapace L Coissieux M-M Wyckoff J Mertz KD Varga Z Junt T. Cessation of CCL2 inhibition accelerates breast cancer metastasis by promoting angiogenesis. Nature (2014) 515:130–3. doi: 10.1038/nature13862 25337873

[121] Haraguchi K Takahashi T Hiruma K Kanda Y Tanaka Y Ogawa S. Recovery of Valpha24+ NKT cells after hematopoietic stem cell transplantation. Bone Marrow Transplant (2004) 34:595–602. doi: 10.1038/sj.bmt.1704582 15300228

[122] Bohineust A Tourret M Derivry L Caillat-Zucman S. Mucosal-associated invariant T (MAIT) cells, a new source of universal immune cells for chimeric antigen receptor (CAR)-cell therapy. Bull Cancer (2021) 108:S92–5. doi: 10.1016/j.bulcan.2021.07.003 34920812

[123] Morandi F Yazdanifar M Cocco C Bertaina A Airoldi I. Engineering the bridge between innate and adaptive immunity for cancer immunotherapy: Focus on γδ T and NK cells. Cells (2020) 9:1757. doi: 10.3390/cells9081757 PMC7464083 32707982

[124] Vivier E Ugolini S Blaise D Chabannon C Brossay L. Targeting natural killer cells and natural killer T cells in cancer. Nat Rev Immunol (2012) 12:239–52. doi: 10.1038/nri3174 PMC5161343 22437937

[125] Godfrey DI Le Nours J Andrews DM Uldrich AP Rossjohn J. Unconventional T cell targets for cancer immunotherapy. Immunity (2018) 48:453–73. doi: 10.1016/j.immuni.2018.03.009 29562195

[126] Sutton KS Dasgupta A McCarty D Doering CB Spencer HT. Bioengineering and serum free expansion of blood-derived γδ T cells. Cytotherapy (2016) 18:881–92. doi: 10.1016/j.jcyt.2016.04.001 27260209

[127] Kitayama S Zhang R Liu TY Ueda N Iriguchi S Yasui Y. Cellular adjuvant properties, direct cytotoxicity of re-differentiated Vα24 invariant NKT-like cells from human induced pluripotent stem cells. Stem Cell Rep (2016) 6:213–27. doi: 10.1016/j.stemcr.2016.01.005 PMC4750166 26862702

[128] Wakao H Yoshikiyo K Koshimizu U Furukawa T Enomoto K Matsunaga T. Expansion of functional human mucosal-associated invariant T cells *via* reprogramming to pluripotency and redifferentiation. Cell Stem Cell (2013) 12:546–58. doi: 10.1016/j.stem.2013.03.001 23523177

[129] Seet CS He C Bethune MT Li S Chick B Gschweng EH. Generation of mature T cells from human hematopoietic stem and progenitor cells in artificial thymic organoids. Nat Methods (2017) 14:521–30. doi: 10.1038/nmeth.4237 PMC5426913 28369043

[130] Montel-Hagen A Seet CS Li S Chick B Zhu Y Chang P. Organoid-induced differentiation of conventional T cells from human pluripotent stem cells. Cell Stem Cell (2019) 24:376–389.e8. doi: 10.1016/j.stem.2018.12.011 30661959 PMC6687310

